# Case of Ovarian Dropsy, Followed by Recovery

**Published:** 1846-04

**Authors:** Richard Eager

**Affiliations:** Surgeon, Guildford


*Case of Ovarian Dropsy, followed by Recovery.* By Richard Eager, Esq., Surgeon, Guildford.—The termination of a case of ovarian dropsy, which lately occurred under my personal observation, is so rare, that I am induced to forward the details of it for publication in the Lancet.

Mrs. ——, aged twenty-eight years, after the birth of her first and only child, now three years old, suffered from attacks of inflammation of the left ovary and phlegmasia dolens in the left leg and thigh, the effects of which left her in an enfeebled state of health for several months.

On the 22d July, 1845, I was consulted by her in reference to a tumour, about the size of a fully developed fœtal head, occupying the left iliac region, easily defined to be circumscribed, moveable, and extending toward the right side, somewhat beyond the median line of the abdomen, having a smooth and even surface, and giving an obscure sense of fluctuation on percussion ; an examination per vaginam manifested a healthy condition of the uterus, although it was somewhat altered in its position, the fundus being thrown forward, whilst its cervix rested on the recto-vaginal septum :—from these symptoms I diagnosed the disease to be encysted dropsy of the left ovarium ; and in this conclusion I was fortified by the opinion of Dr. Lever, who had seen the patient under her former illness, and who was also consulted on the present occasion.

The patient, in the history she gave of her ailment, traced the commencement of it to her recovery from her former illness ; and, as it had made no advance in size, during a period exceeding two years, it was hoped it might continue in a quiescent state. Our attentions were therefore directed to the maintenance of the general health, and the promotion of the healthy functions of the system, by regulated exercise, diet, &c. &c. The compound iodine ointment was used freely over the site of the enlargement, and the hydriodate of potash with a bitter given three times a day.

From this time, however, the disease assumed a more active character, and advanced with such rapidity, that, by the beginning of November, the patient had attained the size of a woman at the full period of gestation, the ovarian tumour occupying the abdominal cavity from the pubes to the ensiform cartilage, producing great inconvenience and distress by its pressure on the diaphragm and surrounding viscera, and tapping was regarded as the only probable means of affording relief.

On the 13th November a purgative was given to relieve constipated bowels,—it induced most distressing and violent vomiting, succeeded by rigors and syncope. On the following day a marked change had occurred in her size and form ; and, to adopt her own expression,—" she felt a sense of want, sinking, and emptiness, almost amounting to fainting." Early on the morning of the 15th November, I was called to her, and found her suffering from all the symptoms of subacute peritonitis, which readily yielded to the usual treatment. On the 17th November a very careful and minute examination of the abdomen failed to detect a vestige of ovarian enlargement, but the whole belly was augmented in size, and over its most dependent parts a very distinct sense of fluctuation was communicated to the hand by percussion. On this day her circumference over the umbilicus was thirty-eight and a half inches. During three or four days subsequent to this date, she evacuated large quantities of urine to the amount of four, five, and six quarts per diem. On the 22d November her size by admeasurement was redneed to twenty-seven inches, with no perceptible enlargement or fluctuation. From the above time no remarkable symptoms have presented themselves, although her convalescence has been retarded by extreme debility :—this has, however, at length yielded to a generous diet, change of air, and a course of " steel bitter" medicine. At the present time she appears to be rapidly advancing to the recovery of her customary good health;—in fact, she told me to-day, she felt better than at any time during the past four years!

From a consideration of the above facts I am induced to believe, that violent action of the abdominal muscles and diaphragm, excited by the vomiting on the 13th November, ruptured the ovarian cyst; and its centents, thus liberated, were extravasated into the abdominal cavity, (producing the attack of peritonitis,) whence they were removed by absorption, the cyst itself remaining in a collapsed and contracted state.—*London Lancet.*

